# Intimate Partner Violence: Using Standardized Patients to Improve Trauma-Informed Care in the era of the Covid-19 Pandemic

**DOI:** 10.15694/mep.2020.000079.1

**Published:** 2020-04-27

**Authors:** Hedda Dyer, Desiree Stelly, Gretchen LeFever Watson

**Affiliations:** 1 Ross University School of Medicine

**Keywords:** Intimate Partner Violence, Domestic Violence, Medical Education, Medical students, Emotional Triggers, Covid-19, Pandemic, Standardized patients, Trauma informed care

## Abstract

This article was migrated. The article was marked as recommended.

Intimate partner violence (IPV) is a global public health problem that has been exacerbated by the social isolation measures currently in place in countries around the world. The authors appreciate the importance of teaching medical students the skill sets to cope with the recognition and diagnosis and medical management of IPV. This is because physicians are most often the first point of contact for victims of IPV. It is also essential to ensure medical students become self-aware of the emotional triggers which may be associated with caring for victims of IPV. This opinion piece explains how medical educators can make a difference in training future physicians in caring for victims of IPV. With the current COVID-19 pandemic bringing the issue of IPV sharply into focus, this paper outlines why medical educators should ensure that medical students are equipped to deal with the societal consequences emanating from the COVID-19 pandemic which will reverberate into the future. Therefore, there is no more time to waste. We are facing a critical juncture, with the current cohort of medical students and physicians exposed to the disproportionately high levels of personal, professional, and emotional trauma that have resulted from the COVID-19 pandemic. Training is imperative; it is of paramount importance for our future medical professionals to be self-aware of their emotional triggers.

## Introduction

“I just never imagined that a 60-year-old woman could be a victim of domestic violence”, was the reason given during a debriefing meeting with a 2nd-year medical student who was unsuccessful at his Clinical exam. And the case scenario on that day was a female who had presented with the chief complaint of “I fell and hit my head,” but who, upon deeper probing was a victim of Intimate Partner Violence (IPV). Intimate partner violence (IPV) refers to abuse and aggression that occurs in a close relationship and is a global public health problem ( World-Health-Organization, 2017). As a surgeon, nurse (RN), and clinical psychologist, we have each encountered patients who have suffered this form of trauma. Both in our clinical roles and as medical educators, we have observed how difficult it can be for many healthcare professionals to adequately appreciate why healthcare professionals should address this common societal problem within the context of a patient encounter. Contrary to common misconceptions, IPV is prevalent in every community and all levels of society, regardless of age, socio-economic status, sexual orientation, gender, race, religion, or nationality. Physical violence is associated with emotionally abusive and controlling behavior, both of which contribute to a systematic pattern of dominance and control. The “devastating consequences of domestic violence can cross generations and last a lifetime.” ( National-Coalition-Against-Domestic-Violence, 2015). IPV has recently been thrust into the international spotlight with the ongoing COVID-19 pandemic. The restriction on movement has caused a plethora of emotional crises for all age groups, genders, and socio-economic statuses. The effect of ‘stay-at-home’ orders has meant that victims of IPV will be isolated with their abusers with little or no way of escape, access, or recourse to help outside the home ( Lanier and Maume, 2009). During this current situation of social isolation, access to healthcare may be the only avenue of escape; a doctor or nurse would be one of the available strategies for victims to escape from the abuse ( Riddell, Ford-Gilboe and Leipert, 2009). There is now an urgent call for action globally to address increased prevalence in IPV resulting from the social isolation measures in which governments around the globe have been enacted ( Bocquet, 2020; Peterman *et al.*, 2020).

## Why does IPV matter to medical educators?

The health effects of IPV are wide-ranging, with a devastating impact on physical, sexual, reproductive, and mental health. IPV can lead to increased morbidity and even death. Thirty-five percent of women worldwide have experienced IPV, and 38 percent of murders committed against women globally are by their intimate partners ( World-Health-Organization, 2017). Physicians are often the first point of contact for victims of IPV; however, physicians are usually not equipped or able to recognize the signs of IPV ( Gotlib Conn *et al.*, 2014). In a small focus group study of Canadian orthopedic surgical residents, many felt unprepared and ill-equipped to interview suspected victims of IPV. They were also uncertain about how and where to refer these patients for help. Despite the high prevalence of IPV, not a single trainee reported learning about IPV during postgraduate training ( Gotlib Conn *et al.*, 2014). Research has also documented that 4th-year medical students feel uncomfortable caring for patients who present with signs and symptoms of IPV ( Heron *et al.*, 2010). There is a need for training of student physicians in recognizing and referring victims of IPV. Now is the opportune time for medical educators to leverage the current pandemic, wherein health care professionals, through their personal experiences of COVID-19 -related trauma, can now view trauma-informed care through the lens of the victim. This conversation is essential to the doctor-patient dyad ( Beverly *et al.*, 2018). Students must be prepared to address the impact of IPV on the physical, sexual, and mental health issues during and after the COVID-19 pandemic.

## How can medical educators make a difference?

As medical school faculty members, and consistent with the published literature, the authors have observed how challenging it is for most medical students to effectively respond to standardized patients who present as victims of IPV. And yet, the Liaison Committee on Medical Education (LCME®) accreditation standards state “the faculty of a medical school ensure that the medical curriculum includes instruction in the diagnosis, prevention, appropriate reporting and treatment of the medical consequences of common societal problems.” (LCME® 2019). Since 2008, this standard has been explicitly tied to the need to address the medical consequences of common societal problems such as IPV. As medical educators, the authors have endeavored to meet the LCME IPV standard by using standardized patients to address the identified IPV training gap. Namely, they have incorporated IPV cases into the 2nd year medical school curriculum as part of the Advanced Interviewing Skills Training (AIST) program. Students’ AIST scores are based, in part, on their ability to recognize and address both the physical and psychological health effects of IPV. To raise awareness that IPV occurs in all iterations of intimate partnerships, the authors are currently piloting a same-sex IPV case. The call to action by the global healthcare community is even more urgent given the ‘new normal’ whereas, as educators, it is incumbent upon us to prepare our students to be self-aware of their own on-going traumatic experiences during the COVID-19 pandemic ( Pfefferbaum and North, 2020), ( Beverly *et al.*, 2018). COVID-19 is the new trigger word and will be so for the foreseeable future. Therefore, as medical educators, we can help our students to prepare for the casualties of COVID- 19 from both a personal and professional perspective, thereby equipping them with the skills sets to practice trauma- informed care.

## Conclusion

With the alarming global IPV prevalence statistics, coupled with their socio-economic and generational impacts on individuals and society, medical educators must improve IPV training among the next generation of physicians. Before graduating from medical school, every physician should be capable of recognizing the signs and symptoms of IPV and skilled at discussing this topic with patients. For maximal results, IPV training ought to begin in undergraduate medical education, before students start their clinical rotations.

## Take Home Messages

In our experience, medical students benefit from having an IPV standardized patient encounter as part of their clinical skills training. We believe that providing this critical component; the medical profession can help to combat the medical consequences of IPV. As has been highlighted by the current COVID-19 pandemic, the call for action is now.

## Notes On Contributors

**Dr. Hedda G. Dyer,** MB CHB Ed, MRCS Ed, MBA is an Associate Professor in Department of Clinical Foundations, Director of the Semester 4X/05 Clinical Skills Course and Clinical Co- Module Director Gastrointestinal Modules at the Ross University School of Medicine, in Barbados. She is a General Surgeon with a Special interest in Breast Surgical Oncology.

**Ms. Desiree Stelly,** MBA, MSN, RN, BSN, BS, CLNCis the Manager of the Standardized Patient Program within the Department of Clinical Foundations, at the Ross University School of Medicine, in Barbados. She is a Registered Nurse.

**Dr. Gretchen B. LeFever Watson,** PhD, is an Associate Professor in the Department of Clinical Foundations at the Ross University School of Medicine in Barbados. She is a Clinical Psychologist and a Consultant for organizational safety and change management.
