# Supplementary material for: Intimate Partner Violence: Using Standardized Patients to Improve Trauma-Informed Care in the era of the Covid-19 Pandemic
Source: MedEdPublish (2016). 2020 Apr 27;9:79. [Version 1] doi: 10.15694/mep.2020.000079.1 (PMC10697487; doi:10.15694/mep.2020.000079.1)
Supplement: Peer review history

# Reviewer report: Reviewer response for version 1

**DOI:** 10.21956/mep.20058.r30765

**Published:** 2020-10-08

**Authors:** Ken Masters

**Affiliations:** 1 Sultan Qaboos University

This review has been migrated. The reviewer awarded 3 stars out of 5 An interesting paper on the very delicate issue Intimate Partner Violence, and using Standardized Patients to improve trauma-informed care in the era of the Covid-19 Pandemic.The authors have succinctly laid out the issues and difficulties, especially under the current isolation, of IPV and teaching medical students about recognising and dealing with it. I would, however, like to see some issues addressed:• “Contrary to common misconceptions, IPV is prevalent in every community and all levels of society, regardless of age, socio-economic status, sexual orientation, gender, race, religion, or nationality”. While this is true, the statement gives the impression that the prevalence is equal across all these demographic groups. It would, perhaps, be useful, to issue a qualifier, with a reference to a source that a reader could consult to see differences.• I would like to see some expansion on the initial statistics: The statistics cited in the heading “Why does IPV matter to medical educators?” are a useful start, but do not complete the picture. Given that physicians are expected to treat people of all genders, and the authors have also noted that IPV occurs in all genders, it would be useful if the statistics of more than one gender could be given, otherwise there is a risk that readers will be alerted to the issue when dealing with one gender only.• Based on the website cited, the percentage cited in this paper appears to be incorrect. The 35% cited includes “non-partner sexual violence”; the figure for IPV is 30%. (I understand that the difference is small, but, especially on such a sensitive topic, it is crucial that the authors are on point with their data).• The title of the paper is a little misleading: The main part of the title is “Using Standardized Patients to Improve Trauma-Informed Care in the era of the Covid-19 Pandemic”. The reader would then expect that the paper is primarily about using standardized patients to improve trauma-informed care in the era of the Covid-19 Pandemic. Yet the description of the relevant project using standardized patients covers barely five lines of the paper (and it is not mentioned in either the Abstract or the Conclusion). I would recommend that either the project is given more space, or the article’s title is adjusted.• A minor point: perhaps the authors could break their paragraphs up a little, so that the reader is not met with a rather imposing wall of text.The project outlined does sound interesting. I look forward to seeing Version 2 of this paper in which further details are given, (and, perhaps later, a more detailed research study on the topic), so that readers may learn more on how to train students around this important topic.

Reviewer Expertise:

NA

No decision status is available

# Reviewer report: Reviewer response for version 1

**DOI:** 10.21956/mep.20058.r30767

**Published:** 2020-05-11

**Authors:** Joshua Francis

**Affiliations:** 1 JFC

This review has been migrated. The reviewer awarded 5 stars out of 5 I have read and cogitated the article under review and hence; Violence by an intimate partner is linked to both immediate and long-term health, social, and economic consequences. Factors at all levels — individual, relationship, community, and societal — contribute to intimate partner violence. This type of violence should include emotional abuse even when partners are not physically isolated together..Mention should have included prevention:What about Preventing intimate partner violence which requires reaching a clear understanding of those factors, coordinating resources, and fostering and initiating change in individuals, families, and society.Usual good job Dr. Dyer and her Team-keep up the good work-relelevant!

Reviewer Expertise:

NA

No decision status is available

# Reviewer report: Reviewer response for version 1

**DOI:** 10.21956/mep.20058.r30764

**Published:** 2020-05-01

**Authors:** Mohamed Hany Shehata

**Affiliations:** 1 Arabian Gulf University

This review has been migrated. The reviewer awarded 1 stars out of 5 I wonder if there is anything new that this article adds. Intimate partner's violance has always been part of students' training and will continue to be.I understand that it is one of multiple problems that will increase because of the current circumstances.The term "standardized patient" simply is not accepted to me at least ethically.

Reviewer Expertise:

NA

No decision status is available

# Reviewer report: Reviewer response for version 1

**DOI:** 10.21956/mep.20058.r30768

**Published:** 2020-04-28

**Authors:** Trevor Gibbs

**Affiliations:** 1 AMEE

This review has been migrated. The reviewer awarded 5 stars out of 5 There is little doubt that the Covid-19 pandemic has caused us to look carefully not only on how we teach, how students learn and our assessment procedures, but it also has caused us to look into our curriculum and think about what is missing. The rise in domestic abuse has been a feature of many recent news items, linking it to the psychological effects of social distancing and quarantining procedures. So this paper has really come at a very important time, pointing out the deficiency of learning outcomes in intimate partner violence (IPV) ( I would include other forms of domestic abuse within this subject). It is a very well written paper that presents a good case for IPV within the curriculum. Of course, including a new subject into what for many is an over-crowded curriculum, means we have to think of what we substitute; that itself means we reflect on the content of our curricula too.I think that this is a very insightful paper that should make curriculum developers consider their curricula, post Covid-19

Reviewer Expertise:

NA

No decision status is available

# Reviewer report: Reviewer response for version 1

**DOI:** 10.21956/mep.20058.r30766

**Published:** 2020-04-28

**Authors:** Samar Ahmed

**Affiliations:** 1 Ain Shams University Faculty of Medicine

This review has been migrated. The reviewer awarded 3 stars out of 5 Thank you for raising this important point that is a rising issue post COVID pandemic. The manifestation of IPV has increased after the lock down and practitioners working in this field have felt this rise. Raising a flag for this issue is really important and the tie between this and the role if medical educators is very important. A great addition to this work was to find an actual guide to support student learning about IPV in this paper. It was kind if frustrating to find no added value for educators who are interested in educating students on IPV. Hopefully more information can be added to sequel papers.

Reviewer Expertise:

NA

No decision status is available
